# Deciphering of *Candida parapsilosis* induced immune response in *Drosophila melanogaster*

**DOI:** 10.1080/21505594.2021.1980989

**Published:** 2021-09-27

**Authors:** Katalin Csonka, Zsolt Tasi, Viktor Vedelek, Csaba Vágvölgyi, Rita Sinka, Attila Gácser

**Affiliations:** a Department of Microbiology, Faculty of Science and Informatics, University of Szeged, Szeged, Hungary; b Department of Genetics, Faculty of Science and Informatics, University of Szeged, Szeged, Hungary; c HCEMM-USZ, Department of Microbiology, Faculty of Science and Informatics, University of Szeged, Szeged, Hungary; d MTA-SZTE Lendület Mycobiome Research Group, University of Szeged, Szeged, Hungary

**Keywords:** *Candida*, innate immune response, virulence, recognition, systemic infection, *Drosophila melanogaste*, cell wall, β-glucan

## Abstract

*Candida* infections are the most prevalent cause of serious human mycoses and are the third most common pathogens isolated from bloodstream infections in hospitalized patients. *C. parapsilosis* is a member of the *non-albicans* spp., which have a predilection for causing life-threatening disease in neonates and hospitalized pediatric patients. In this study, we utilized a *Drosophila melanogaster* infection model to analyze the immunological responses to *C. parapsilosis*. Our results demonstrate that the Toll pathway in *Drosophila* controls *C. parapsilosis* proliferation as the Toll signaling mutant *MyD88^−/^*^−^ flies are highly susceptible to *C. parapsilosis*. We also confirmed that the *MyD88^−/^*^−^ fly is a convenient invertebrate animal model to analyze virulence properties of different species and strains from the *C. parapsilosis sensu lato* complex as *C. orthopsilosis, C. metapsilosis* proved to be less virulent than *C. parapsilosis sensu stricto* and the *N*-mannan deficient *C. parapsilosis och1*Δ/Δ strain showed attenuated pathogenicity in this immunodeficient *Drosophila* background. We also found that Persephone protease is not required for detection and activation of Toll pathway during *C. parapsilosis* infection. Furthermore, we observed that *Drosophila* β-glucan receptor deficient flies where more sensitive to *C. parapsilosis* compared to wild-type flies; however, we could not find a clear dependence on the recognition of this receptor and the cell wall β-glucan exposure-induced host response. These studies establish this *D. melanogaster* infection model as an efficient tool in deciphering immune responses to *C. parapsilosis* as well as for assessing virulence factors produced by this emerging fungal predator.

## Introduction

*Candida* species are opportunistic fungal pathogens causing severe diseases in immunocompromised patients and are the third most common microorganisms responsible for healthcare-related bloodstream infections [1]. Although *C. albicans* is the most frequently isolated species globally as the causative agent for disseminated *Candida* diseases, the frequency of infections due to non-*albicans Candida* species continues to increase [2,3]. *C. parapsilosis* is ranked as the second or third most common non-*albicans* spp [4]. This pathogen has a particular predilection for causing hospital-acquired infections in neonatal and pediatric patients as well as adult patients with intravascular catheters and other implantable devices [5,6]. Through investigations of *C. parapsilosis* biology, numerous factors have been identified that play roles in pathogenesis including extracellular lipases, transcription regulators, pseudohyphae and biofilm production, antifungal resistance mechanisms, and iron metabolic processes [6–8].

Since virulence-related genes in various *Candida* spp. require validated models to define their function, experimental *in vivo* models are essential in pathogenesis research. The fruit fly, *Drosophila melanogaster* is a remarkably flexible invertebrate model organism to study specific responses of innate immunity against microbial infections [9,10]. This mini-host has been applied to examine innate immune defense mechanisms against certain *Candida* species, as flies deficient in the Toll signaling pathway are sensitive to fungal infections [11]. The Toll/Dif pathway responds to the presence of fungal and Gram-positive bacterial infections and mediates the production of antimicrobial peptides (AMPs), such as Drosomycin, Metchnikowin, and Defensins [12–14]. After it is activated by a proteolytic cleavage cascade, Spätzle (*spz*) is a ligand for Toll and binds to the cell transmembrane receptor, triggering its dimerization, which leads to the recruitment of the adaptor, Drosophila myeloid differentiation factor 88 (*dMyD88*, homolog of mammalian MyD88). Upstream of Spätzle, immune detection of fungal determinants is regulated by the Gram-negative binding protein 3 (*GNBP3*) and the Persephone (*psh*) serine protease [15]. The GNBP3, a member of the GNBP/β-glucan recognition proteins (βGRP) family, has been reported to bind to fungal cell wall β-(1,3)-glucan and activates the antifungal Toll pathway in a Spätzle-dependent manner [15]. Indeed, GNBP3 contributes to controlling *Candida* infections, as *C. albicans* and *C. glabrata* challenged *GNBP3* deficient flies display increased susceptibility/death events and impaired expression levels of the Toll-dependent *Drosomycin* gene [15,16]. *Psh* encodes a hemolymphatic serine protease belonging to a *Drosophila* danger pathway and becomes activated by proteolytic activities of microbes that induce Toll signaling [15]. The lack of *psh* causes a weak susceptibility of adult flies to *C. albicans*, but *psh* mutant flies are highly susceptible to *C. glabrata* challenge [16,17]. The contribution of these sensor molecules in *Candida* defense can vary depending on the *Drosophila* model selected. For example, gastrointestinal infection with *C. albicans* in *Drosophila* larvae generated a GNBP3 independent, but psh-Toll dependent systemic response, which required the presence of hemocytes [18].

A broad range of research has demonstrated that *Drosophila* models are reliable tools for screening new antifungal treatment options against *C. albicans* [11] and *C. auris* [19] and investigating genes involved in *Candida* pathogenesis. Different virulence factors, such as *C. albicans* Cas5, a transcriptional regulator of genes involved in cell wall integrity [20] and secreted aspartyl proteases SAP4 and SAP6 [18], as well as *C. glabrata* Yapsins (secreted GPI-anchored aspartyl proteases) [16] and ADA2 for oxidative stress tolerance were identified in this mini-host [21].

In this study, we aimed to describe *C. parapsilosis* infection in an immunodeficient *D. melanogaster* fly model, which we adapted from the work previously performed to characterize *C. albicans* and *C. glabrata* induced specific immune responses [16]. We demonstrated that *C. parapsilosis* infection is highly regulated by the *Drosophila* Toll pathway, as *MyD88* mutant flies succumbed to challenge with *C. parapsilosis* cells. We extended our studies to include additional members of the *C. parapsilosis sensu lato* complex, and demonstrated that this type of immunodeficient fly is suitable to analyze the differences in virulence of the *C. parapsilosis sensu lato* complex strains. We also found, as reported in mouse models, that a *C. parapsilosis* without a proper *N*-mannan layer in the cell wall was significantly less virulent in our fly model. Our results show that *D. melanogaster* and mutants like *MyD88^−/-^ Drosophila* are extremely useful model for identifying and analyzing *C. parapsilosis* virulence factors.

### Materials and methods

#### Drosophila stocks

*Drosophila* stocks were maintained on standard cornmeal agar medium at 25°C, in 12 h light/12 h dark cycle according to *Drosophila* Protocols, Chapter 35 (Sullivan, Ashburner, Hawley, Cold Spring Harbor Laboratory Press, 2000). The wild-type *w^1118^* (BL3605) (from Bloomington Stock Center) and Drosomycin-GFP (GFP-Drs-fly) [22], *GNBP3^hades^, psh*, and *MyD88* mutant flies (a kind gift from Jessica Quintin) were used in this study. Stocks have been described previously [23].

#### Microbial strains

*C. parapsilosis* GA1 (SZMC 8110) [24], *C. parapsilosis* CLIB 214 (SZMC 1560) [25] *C. parapsilosis* SZMC 1592, *C. parapsilosis* SZMC 8050, *C. albicans* SC5314 (SZMC 1523), *C. metapsilosis* SZMC 1548, *C. metapsilosis* SZMC 8099, *C. metapsilosis* SZMC 8094, *C. orthopsilosis* SZMC 1545, *C. orthopsilosis* SZMC 8121 and *C. orthopsilosis* SZMC 8119 [26] wild-type strains, *C. parapsilosis och1*Δ/Δ, *C. parapsilosis* CPRI [27], a GFP-expressing derivative of *C. parapsilosis* CLIB 214 (genotype: CpNEUT5L/CpNEUT5L::pECpOE-GFP-N-N5L) and *C. albicans* SC5314 (RPS1/RPS1::CIp10-P_TDH3_-GFP-CaNAT1) were used in this study and maintained on YPD agar plates (0.5% yeast extract, 1% peptone, 1% glucose, 2.5% agar) at 4°C. The *C. parapsilosis* CPRI strain was used as a reference for the analysis of infections with *C. parapsilosis och1∆/∆* strain. Prior to use, *Candida* cells were grown in liquid YPD medium (0.5% yeast extract, 1% peptone, and 1% glucose supplemented with 1% Penicillin-Streptomycin) with shaking (200 rpm) at 30°C overnight. *Micrococcus luteus* SZMC 0264 (Szeged, Hungary), a Gram-positive bacteria, was used as a reference for the *Drosomycin* induction studies. *M. luteus* was grown in an overnight culture in LB broth (1% tryptone, 0.5% yeast extract and 0.5% NaCl) at 37°C with 200 rpm shaking. Prior to use, microbial cells were harvested by centrifugation, washed twice with PBS (phosphate-buffered saline; 137 mM NaCl, 2.7 mM KCl, 10 mM Na2HPO4, 2 mM KH2PO4; pH 7.4), counted using a hemocytometer and adjusted to the proper concentration detailed for each experiment.

#### Survival study of flies

Batches of 15 (2- to 4-day-old females and males; 45 per experimental group) wild-type (wt) and mutant flies were infected by septic injury on the dorsal side of the thorax. The flies were injected using a 30 g needle previously dipped into PBS or a 2×10^7^/ml suspension of bacteria or yeasts. The vials containing the challenged flies were housed in an incubator (29°C for fungal infections or 25°C for bacterial challenges). Survival was assessed daily, and live flies were put into new vials containing standard cornmeal agar medium every second day. Results are expressed as a percentage of surviving flies at different days post-infection.

Even though it may not be ideal for examining the virulence of human pathogens at temperatures below 37°C, the incubation of flies at 29°C is a good compromise to avoid the physiological consequences of the heat-shock response. We choose this methodology according to our experiences and previous work of Davis et al., where they demonstrated that performance of *Drosophila*-fungus interaction at 29°C is suitable for examining *C. albicans* virulence factors and this temperature has no adverse effect on the yeast dissemination and the development of pseudohyphae and hyphae [28].

#### Fungal burden assay

Groups of 10 infected flies were homogenized in PBS at specific times (right after the infection (input), 5 hours (0 day), 2 days, and 4 days) after the PBS and *Candida* (2×10^7^/ml) infection. The homogenates were serially diluted, plated on YPD agar plates, and incubated for 48 h at 30°C to enable colony growth for counting. Yeast colonies recovered from flies were calculated and expressed as CFU/fly. Results are pooled data from five independent experiments.

#### In vivo phagocytosis assay

Flies were infected with 20 μl of a 1×10^5^/ml GFP-labeled *Candida* strain suspension using a sharpened glass capillary on the thorax and then the insects were incubated for 3 h at 25°C. Collection of hemocytes was performed according to a standard method [29]. Briefly, flies were anaesthetized and the last section of the abdomen was removed. The fly’s thorax was punctured with a sharpened glass capillary. Perfusion was performed through a capillary with a Schneider’s medium (Biowest, cat.: L0207) containing 1-phenyl-2-thiourea (PTU, Sigma-Aldrich, cat.: P7629). The samples collected from five flies per group were placed on glass slides and incubated for 30 min to allow hemocytes to adhere to the slides. After the incubation, the medium was removed, and non-phagocytosed yeasts were labeled with 5 μM of Calcofluor white (5 mM, Sigma-Aldrich, cat.: 18,909–100ML-F) at room temperature for 10 min and then washed two times with PBS to remove excess stain. Samples were fixed for 5 min in 4% formaldehyde in PBS, permeabilized for 5 min in 0.1% Triton X-100 and filamentous actin of *Drosophila* hemocytes was stained with Texas Red®-X Phalloidin (Thermofisher, cat.: T7471) (1:250) for 20 min. After washing steps with PBS, samples were covered with SlowFade mounting medium (Invitrogen, cat.: S36917) and the slides analyzed with a BX51 OLYMPUS microscope.

#### RNA isolation and qPCR

The measurement of *Drosomycin* mRNA level was designed and performed according to a standard method [30]. Samples of five flies/group were frozen in liquid nitrogen and total RNA was isolated using the Quick-RNA MiniPrep Kit (Zymo Research, cat.: R1054) according to the manufacturer’s instructions. The concentration and integrity of isolated RNA were confirmed by ND-1000 Spectrophotometer (Thermo Scientific). cDNA was synthesized from 2000 ng total RNA using the RevertAid^TM^ First Strand cDNA Synthesis Kit (Thermo Scientific, cat.: K1622) according to the manufacturer’s protocol. qRT-PCR was performed using Maxima SYBR Green qPCR Master Mix (Thermo Scientific, cat.: K0242), in a C1000^TM^ Thermal Cycler (BIO-RAD) equipped with a CFX96™ Real-Time Detector System (BIO-RAD). *Ribosomal protein 49* (*Rp49*) was used as an endogenous control gene, and fold changes were calculated by the ΔΔC_t_ method. PCR product specificity was confirmed by melting analysis. Primer sequences were as follows: *rp49*: forward: 5’ GACGCTTCAAGGGACAGTATCTG 3’, reverse: 5’ AAACGCGGTTCTGCATGAG 3’; *drosomycin*: forward 5’ CGTGAGAACCTTTTCCAATATGATG 3’, reverse: 5’ TCCCAGGACCACCAGCAT 3’ [30].

#### Detection of Drosomycin production by microscopy

GFP-Drs-flies were injected with 2×10^8^/ml *Candida* or bacterial suspension [22]. After 24 h of incubation, flies were anaesthetized for direct observation of Drosomycin induction. Microscopy was performed using an OLYMPUS SZX7 stereomicroscope.

#### Statistical analysis

Graphs represent at least three independent experiments (*n* ≥ 3 in each experiment) that yielded similar results unless otherwise stated (see Results and Figure legends for details). Results from the fungal burden and real-time PCR analysis are expressed as mean ± SEM. Diagrams were created and statistical analyses were performed with the GraphPad Prism 7.0 software. Differences were considered statistically significant at *p* ≤ 0.05.

## Results

### Toll pathway involvement in Candida infection

**Figure 1.**
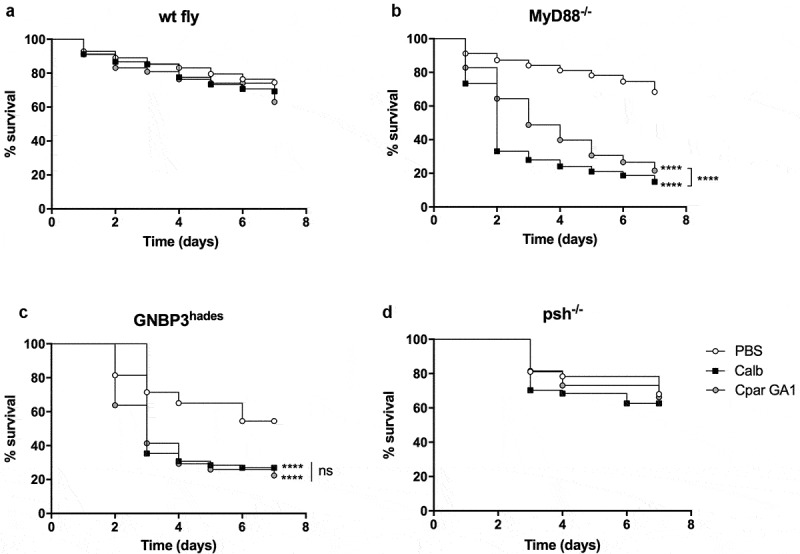
Comparison of the survival of wt (a), *MyD88^−/-^* (b), *GNBP3^hades^* (c) and *psh^−/-^* (d) flies after injection with PBS, *C. albicans* SC5314 or *C. parapsilosis* GA1. Infection dose 2 × 10^7^ yeast/ml. n = 45 fly/group/experiment. Results are representative of 4 independent experiments with statistical analysis by Mantel–COX test. P value style: GP: **** p < 0,0001; not significant (ns)<0.1234

To determine if *D. melanogaster* are susceptible to *C. parapsilosis* and whether the Toll signaling pathway regulates disease, we injected wild-type (wt fly) and *MyD88^−/-^* flies with *C. parapsilosis* GA1 or *C. albicans* SC5314 strain, as reference. As expected, there was no significant difference in survival rates of wt flies infected with the fungal species compared to the PBS injected flies (Figure 1(a)). Notably, *MyD88^−/-^* flies displayed similar susceptibility to *C. albicans* and *C. parapsilosis* GA1. However, with a more in-depth examination of the mini-host’s survival, we observed that more *MyD88^−/-^* flies challenged with *C. parapsilosis* survived compared to *C. albicans* infected flies (Figure 1(b)). The lower virulence of *C. parapsilosis* in the *Drosophila* model was previously reported by Chamilos et al., where the *Tl* mutant flies died more from *C. albicans* or *C. krusei* than *C. parapsilosis* [11]. Therefore, our data further strengthened the observation that the Toll pathway is required for defense against these opportunistic yeasts and the *Drosophila* model is suitable to distinguish between the different virulence potentials of distinct fungal species.

To detail the upstream events participating in the Toll pathway activation against *C. parapsilosis*, we also tested flies carrying a mutation in the GNBP3 receptor or the Persephone serine protease. During the monitoring of GNBP3 deficient flies’ survival, we noted that the absence of GNBP3 PRR markedly affected the fly’s fitness and viability, as the PBS injection alone caused death events in our experimental settings. As expected from the previous studies, *GNBP3^hades^* flies nevertheless showed increased susceptibility to *C. albicans* compared to the PBS injected fly groups. Our data also documents that this receptor takes part in detecting *C. parapsilosis* cells, as the *GNBP3^hades^* flies displayed increased mortality in response to this pathogen compared to the PBS injected fly group (Figure 1(c)). Furthermore, we observed that *psh^−/-^* flies were resistant to *C. parapsilosis* and *C. albicans* (Figure 1(d)).

### Effect of Candida infection on antimicrobial peptide gene expression

Upon fungal challenge, the *Drosophila* pathogen recognition receptors trigger signaling pathways leading to the production of the antifungal peptide Drosomycin. *Drosomycin* mRNA-level measurement has been used as a readout of Toll pathway activation, and its induction was reported upon *Candida* infections [15]. Therefore, we challenged a transgenic *Drosophila* line expressing the GFP-Drosomycin fusion protein [22] to examine the response induced by *C. parapsilosis*. We used *M. luteus* as bacterial [31] and *C. albicans* as fungal reference. As survival of wt, *MyD88^−/-^* and *GNBP3^hades^* flies showed no significant difference between infection with *C. parapsilosis* GA1, *C. parapsilosis* CLIB 214 and *C. parapsilosis* CPRI strains (Figure S1(a-c)), we presented data performed with *C. parapsilosis* CPRI strain.

**Figure 2.**
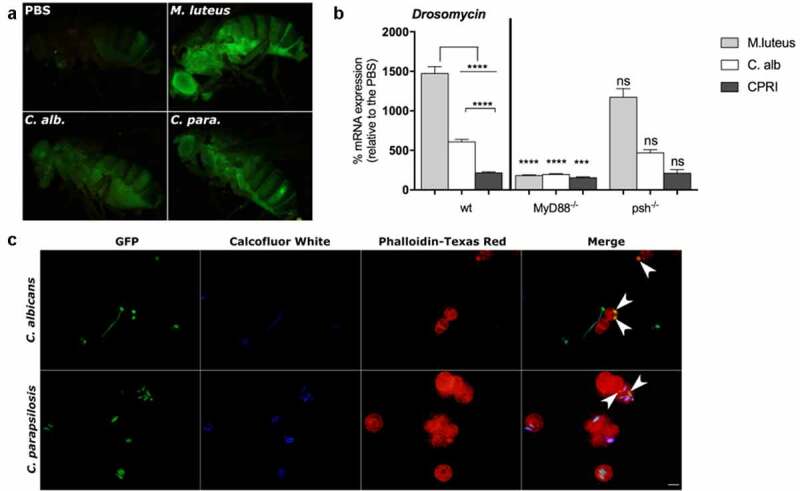
A. GFP-Drosomycin expression of flies after 24 h of injection with *M. luteus, C. albicans* or *C. parapsilosis* CPRI. Injection dose 1x10^8^/ml. B. *Drosomycin* mRNA induction in wt, *MyD88^−/-^* and *psh^−/-^* flies after 24 h of injection with *M. luteus, C. albicans* (C. alb) or *C. parapsilosis* CPRI. Injection dose 5x10^7^/ml. Data are represented as means with ± SEM from 3 independent experiments as determined by paired t-test. P value style: GP: **** p < 0,0001; *** p < 0,0002; not significant (ns)<0.1234. C. *In vivo* phagocytosis of GFP-*C. albicans* and GFP-*C. parapsilosis*. Flies (wt) were injected with 20 µl of 2×10^5^/ml of GFP-*C. albicans* or GFP-*C. parapsilosis* strains. Hemolymph was collected (5 fly/group) 3 h after the injection, non-phagocytosed yeast were labeled with Calcofluor White, and hemocytes were stained with Phalloidin-Texas Red. White arrows indicate engulfed *Candida* cells

As expected, *M. luteus* injected flies exhibited a robust GFP-expression compared to the *Candida* infected fly groups (Figure 2(a)). In comparison to the PBS injected fly, *C. albicans* and *C. parapsilosis* challenged flies presented a strong GFP-Drosomycin expression, suggesting that systemic infection with either of the two *Candida* species similarly induces the production of this AMP in the fruit fly (Figure 2(a)).

To depict the activation of the humoral response against *C. parapsilosis*, we measured the mRNA level of *Drosomycin* using quantitative real-time PCR method in the wt, *MyD88^−/-^* and *psh^−/-^* flies. In the *GNBP3^hades^* fly, PBS injection alone caused higher *Drosomycin* mRNA expression in this background than the wt fly groups (Figure S1(d)); therefore, we did not include this fly strain in our analyses. After 24 h of the infection, wt flies showed the highest expression of AMP after challenge with *M. luteus,* whereas the yeast species induced lower mRNA levels. Furthermore, *C. parapsilosis* provoked a significantly weaker humoral response compared to *C. albicans* (Figure 2(b)). In comparison to the wt fly, a mutation in the MyD88 adapter resulted in a significantly decreased level of *Drosomycin* after either the bacterial or fungal stimuli. Similarly, as reported for *C. albicans* [15,16], antifungal peptide gene induction by *C. parapsilosis* was not affected by lack of Persephone protease, as no differences were detected in its mRNA levels between the corresponding *psh^−/-^* and wt fly groups (Figure 2(b)).

These data confirmed the results presented by the survival experiments and indicate that *C. parapsilosis* infection cause Toll-mediated humoral defense in *Drosophila*.

### Detection of phagocytosis upon Candida infection

We next examined *in vivo* phagocytosis, which is one of the cellular responses of the *Drosophila* immune system. For this, we infected adult flies by septic injury with a suspension of *C. albicans* or *C. parapsilosis*, and examined the phagocytosis capacity of hemocytes against the yeast cells. After 3 hours of incubation, we found that *Drosophila* blood cells effectively detected and engulfed both *C. albicans* and *C. parapsilosis* cells. Representative pictures show phagocytosed yeast cells of *C. albicans* and partially enveloped cells of *C. parapsilosis* (Figure 2(c)). These results confirmed a systemic response after *C. parapsilosis* septic wounding and suggest a similar elimination mechanism against this yeast as to that described with *C. albicans* in *Drosophila* [32].

### Assessment of virulence properties of different C. parapsilosis strains

**Figure 3.**
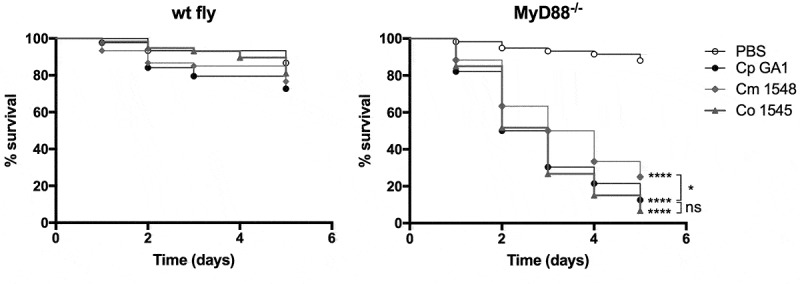
Survival of wt, *MyD88^−/-^* flies after injection with PBS, *C. parapsilosis* GA1, *C. metapsilosis*, or *C. orthopsilosis*. Infection dose 2 × 10^7^ yeast/ml. n = 45 fly/group/experiment. Results are representative of 4 independent experiments with statistical analysis by Mantel–COX test. P value style: GP: **** p < 0,0001; * p < 0,0332; not significant (ns)<0.1234

As our results showed that *MyD88^−/-^* flies are susceptible to *C. parapsilosis* challenge, we wanted to examine whether this fly group could be utilized to determine virulence differences in closely related *C. parapsilosis sensu lato* complex species. Therefore, we injected the wt and the *MyD88^−/-^* flies with three isolates each of *C. parapsilosis, C. orthopsilosis* or *C. metapsilosis* species, and found that the *MyD88^−/-^* flies display increased susceptibility to the members of the *C. parapsilosis sensu lato* group compared to the PBS injected fly group (data not shown). As next step, we selected one isolate of *C. parapsilosis* (Cp GA1), *C. orthopsilosis* (Co 1548) and *C. metapsilosis* (Cm 1546) and compared their virulence in the immune-deficient mini-host. In agreement with previous studies with another invertebrate model, *Galleria mellonella* larvae [26], our results presented that the *C. metapsilosis* infected *MyD88^−/-^* flies had significantly better survival rates than flies challenged with the other *C. parapsilosis sensu lato* species. Furthermore, no significant differences were detected in the death events caused by the *C. parapsilosis sensu stricto* and *C. orthopsilosis* challenged *MyD88* deficient flies (Figure 3).

### Effect of C. parapsilosis cell wall integrity on pathogenesis in the D. melanogaster model

Next, to characterize the pathogenesis of *C. parapsilosis* in *D. melanogaster* and test whether this invertebrate model is suitable to assess differences in the virulence of mutant *C. parapsilosis* strains, we employed the *C. parapsilosis* CPRI reference and the mutant *och1Δ/Δ* (*Cpoch1∆/∆*) for fly injection. The *C. parapsilosis och1∆/∆* strain exhibits a severe defect in *N*-mannan content with elevated β-glucan and chitin levels in the cell wall. In previous studies, *Cpoch1∆/∆* strain-induced alterations in the cytokine production in human mononuclear cells and displayed significantly decreased virulence in Balb/C mouse and neonate mouse model [27,33].

**Figure 4.**
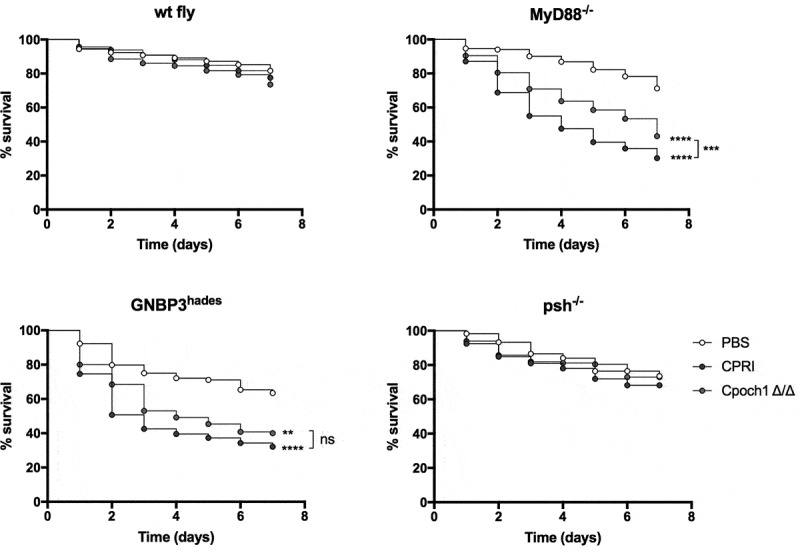
Survival of wt, *MyD88^−/-^, GNBP3^hades^* and *psh^−/-^* flies after injection with *C. parapsilosis* CPRI or *Cpoch1∆/∆*. Injection dose 2×10^7^/ml. Results are representative of 4 independent experiments with statistical analysis by Mantel-Cox-test. P value style: GP: **** p < 0,0001; *** p < 0,0002; ** p < 0,0021; * p < 0,0332; not significant (ns)<0.1234

In agreement with the findings of systemic murine infection, our results revealed that the percentage of surviving *MyD88^−/-^* flies were significantly higher after challenged with the *Cpoch1∆/∆* cells compared the *C. parapsilosis* CPRI infected fly groups (Figure 4). In comparison to PBS, the *GNBP3^hades^* flies died significantly faster after challenge with the cell wall mutant *Candida* strain, but mortality rate was similar to that caused by *C. parapsilosis* CPRI. As expected, the *psh* mutant flies were resistant to *Cpoch1∆/∆* infection (Figure 4).

**Figure 5.**
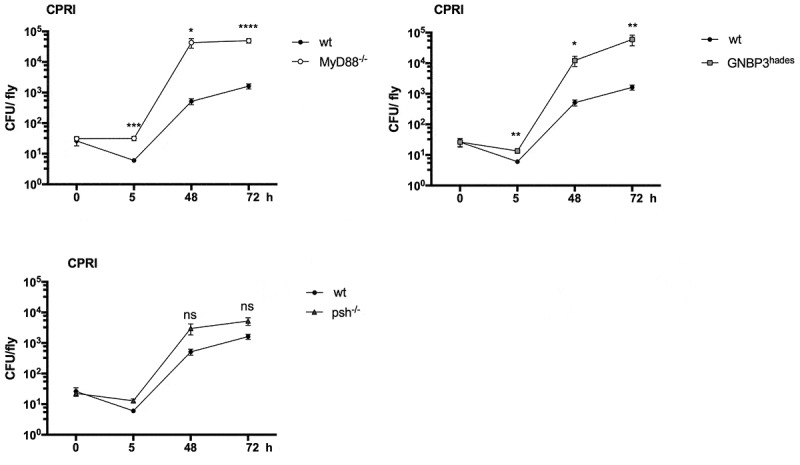
CFU assessment of wt, *MyD88^−/-^, GNBP3^hades^* and *psh^−/-^* flies after injection with *C. parapsilosis* CPRI. Injection dose 2×10^7^/ml. Data are presented as mean with ± SEM from 5 independent experiments as determined by paired t-test. P value style: GP: **** p < 0,0001; *** p < 0,0002; ** p < 0,0021; * p < 0,0332; not significant (ns)<0.1234

For a detailed assessment of the two *C. parapsilosis* strain’s virulence properties, we analyzed the proliferation capacity of the fungi in flies using CFU determinations. As shown, the wt flies were resistant to *C. parapsilosis* infection, and the CFU results show that these insects can rapidly kill *C. parapsilosis* CPRI cells. However, *C. parapsilosis* CPRI that survived the initial immune response were able to proliferate in these flies, as increased numbers of yeast cells were observed during the infection period (Figure 5) The susceptibility of the *MyD88^−/-^* and *GNBP3^hades^* flies to *C. parapsilosis* infection was also strengthened by the significantly higher fungal loads at 2 and 4 days of infection compared to that observed in wt flies. The assessment of the colonization also supported the resistance of *psh* mutant fly. The *psh^−/-^* flies showed that the yeast cells could survive within the mini-host, but the fungal loads were relatively low (Figure 5).

**Figure 6.**
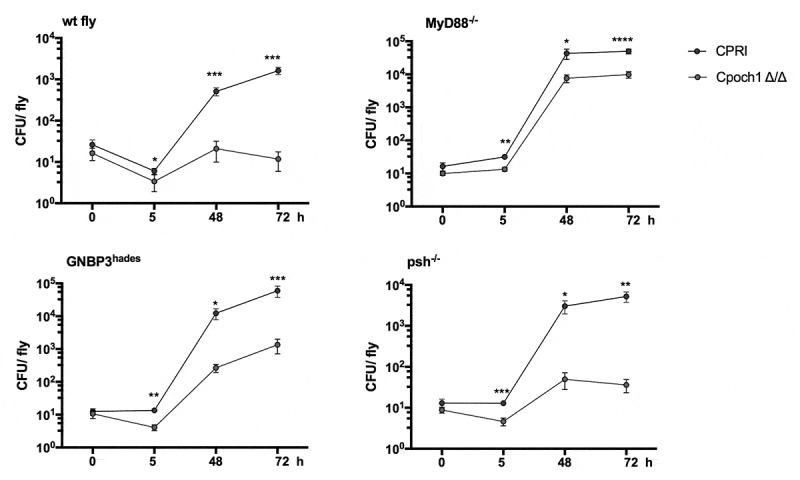
CFU assessment of wt, *MyD88^−/-^, GNBP3^hades^* and *psh^−/-^* flies after injection with *C. parapsilosis* CPRI and *Cpoch1∆/∆*. Injection dose 2×10^7^/ml. Data are represented as mean with ± SEM from 5 independent experiments, Paired t-test. P value style: GP: **** p < 0,0001; *** p < 0,0002; ** p < 0,0021; * p < 0,0332

Notably, we detected a decrease in fungal colonies of *Cpoch1∆/∆* infected wt and *psh^−/-^* flies from day 2 to day 4, suggesting an enhanced clearance of the mutant *C. parapsilosis* strain by the mini-host (Figure 6). In line with the survival data, as the *Cpoch1∆/∆* challenged *MyD88^−/-^* and *GNBP3^hades^* flies showed an increment in the death events compared to the PBS injected fly groups, these genotypes of flies were unable to clear the *Cpoch1∆/∆* cells. However, all groups of flies, either sensitive to the reference *C. parapsilosis* strain or not, were able to control the growth of the *N*-mannan mutant strain, as significantly lower CFUs of *Cpoch1∆/∆* were obtained from each fly background at each time point of the experiments compared to *C. parapsilosis* CPRI (Figure 6). Therefore, these CFU data also support the decreased virulence of *Cpoch1∆/∆* in the immune-deficient *Drosophila* model.

**Figure 7.**
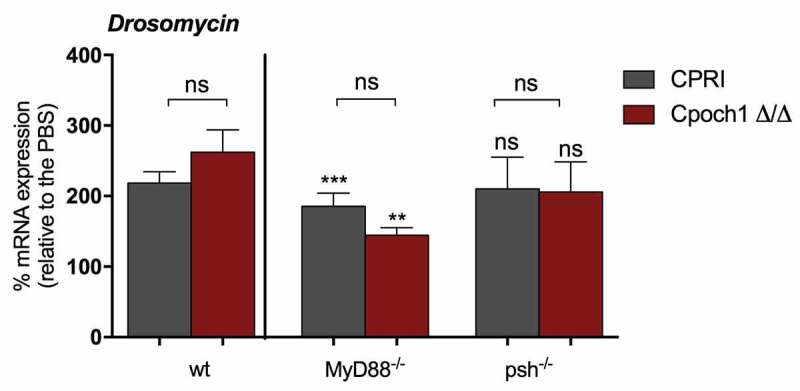
*Drosomycin* mRNA induction in wt, *MyD88^−/-^* and *psh^−/-^* flies after injection with *C. parapsilosis* CPRI or *Cpoch1∆/∆*. Injection dose 5x10^7^/ml. Data are presented as means with ± SEM from 3 independent experiments as determined by paired t-test. P value style: *** p < 0,0002; ** p < 0,0021; not significant (ns)<0.1234

Next, we tested whether the decreased virulence of the *Cpoch1∆/∆* is commensurate with its induction of antifungal peptides. In comparison to the *C. parapsilosis* CPRI, *Cpoch1∆/∆* induced a non-significant increase in *Drosomycin* mRNA level in the wt fly (Figure 7). When the *MyD88^−^*^/-^ flies were infected with the *Cpoch1∆/∆*, we found significantly decreased mRNA level of the antifungal peptides following challenge with CPRI or *Cpoch1∆/∆* compared to levels in wt flies, but there were again no significant differences between the *C. parapsilosis* strains. *Drosomycin* expression measured from *psh^−/-^* flies infected with either *C. parapsilosis* strain were similar to levels in wt insects (Figure 7). Therefore, these data further strengthened the results that the Toll pathway detects *C. parapsilosis* cells and the Persephone serine protease is not involved in its activation process.

## Discussion

Here, we aimed to use *D. melanogaster* as a model to investigate the pathogenicity of *C. parapsilosis*. Our results indicate that the *Drosophila* Toll restrains *C. parapsilosis* proliferation as *MyD88^−/^*^−^ flies display a significantly enhanced susceptibility to *C. parapsilosis*. Our data also support an earlier study where *C. parapsilosis* showed lower virulence in *D. melanogaster* compared to *C. albicans* [11]. We have further explored the capacity of this mini-host to sense *C. parapsilosis* using flies lacking the GNBP3 β-glucan receptor or the Persephone protease required for Toll pathway activation during fungal invasion. We found that *GNBP3^hades^* flies displayed increased susceptibility to *C. parapsilosis*, whereas *psh* mutants were resistant, which is similar to findings with *C. albicans* challenge in these fly strains [15]. Furthermore, we demonstrated that the *MyD88^−/-^ Drosophila* strain could distinguish variations in virulence between the closely related *Candida* species, and that the characterizations were similar to that found in our prior work using a *G. mellonella* model where *C. metapsilosis* was the least virulent species of the *psilosis* group and no significant divergence was observed between the mortality rate of larvae infected with *C. parapsilosis sensu stricto* and *C. orthopsilosis* isolates [26].

Our study examined whether immune-deficient flies might be useful to identify and test the variation of *C. parapsilosis* strains’ pathogenesis. The α1,6-mannosyltransferase Och1 initiates *N*-glycan outer chain branch addition in the yeast cell wall and possibly regulates virulence in both *C. albicans* and *C. parapsilosis* [27,34]. In the *MyD88* mutant *D. melanogaster*, the lack of *N*-mannan content in *C. parapsilosis* altered the survival rates of infected flies compared to insects infected with the reference yeasts. The decreased virulence of *C. parapsilosis* lacking *N-*mannan in this mini-host is similar to results found in studies using a systemic mouse infection model [27,33]. Albeit, the decreased virulence with this *C. parapsilosis* mutant was not paired with differences in antimicrobial peptide induction in the fly. A similar result was found in a gastrointestinal *Drosophila* larvae model where the *C. albicans* cell wall mutant *PMR1*, which has defects in both *N*- and *O*-linked mannosylation, activated *Drosomycin* to the same extent as did the wild-type *C. albicans* counterpart strain [18].

*C. parapsilosis* induced *Drosomycin* at a significantly lower rate than *C. albicans* in wt and the *psh^−/-^* flies, and the flies were more resistant to *C. parapsilosis*. Also, the *Cpoch1Δ/Δ* strain demonstrated attenuated virulence in the *Drosophila* model, but it induced an antimicrobial response that was similar to the reference *C. parapsilosis* strain. There are controversial results regarding the fungicidal activity of Drosomycin against yeasts. *In vitro* studies noted that Drosomycin has no fungicidal effect on *C. albicans* and *C. glabrata* [16,35,36]. A study using a knockout approach of different AMPs deficient flies deduced that AMPs have may not individually be essential in defense against fungi and disclosed the additive cooperation of *Drosomycin* and *Metchnikowin* to restrain *C. albicans* infection [37].

Thus, our results indicate that it is not primarily the antimicrobial peptide production that performs the elimination function in *Drosophila* in controlling *C. parapsilosis* infection as it was correspondingly concluded for *C. albicans* and *C. glabrata* [38].

GNBP3 is essential for controlling *C. albicans* and *C. glabrata* infections, as deprivation of this receptor caused increased susceptibility of adult flies against these *Candida* species [16,32]. Unexpectedly, in our experiment settings, the *GNBP3^hades^* flies were extremely sensitive to injection as the survival proportion of the PBS treated flies was around 56%. However, the death events of the *C. albicans* or *C. parapsilosis* challenged fly group were significantly higher compared to the PBS injection. We were surprised that *och1Δ/Δ* and the reference *C. parapsilosis* strain infection provoked similar survival curves in the GNBP3 deficient flies, albeit the wild-type produced significantly hihger CFUs compared to the mutant. Therefore, we could not find clear interdependence between the lack of the *N*-mannosyl residues and the higher β-glucan exposure in the cell wall of *C. parapsilosis* and ligand binding of this *Drosophila* receptor.

We also found elevated *Drosomycin* mRNA levels in *GNBP3^hades^* flies compared to the wild-type *Drosophila* after the PBS injection alone. This could suggest that the death events of the *Candida*-challenged GNBP3 receptor mutant flies were not necessarily the sole effect of the fungus, but the deficiency of this receptor could cause the lack of some specific response to the injury. Therefore, the combined effect of the fungus and the infection route may generate the phenomenon that no difference was detected between the survival of the *C. albicans*- and the *C. parapsilosis* strains-challenged *GNBP3^hades^* fly groups. Results from gastrointestinal infection of *Drosophila* larvae also registered that absence of GNBP3 receptor did not influence systemic activation of *Drosomycin* and double mutant *psh; GNBP3* larvae exhibited a similar decrease in the level of the antimicrobial peptide as *psh* mutants following infection with live *C. albicans* [18]. This study suggests the altered immune sensing processes, including the role of GNBP3 between the larvae and adult fly and the *Drosophila* gut and systemic infection model. The mammalian β-glucan receptor, Dectin-1, displays a similar feature. In mice, Dectin-1 is indispensable in regulating systemic infection with *C. albicans,* but it performs a redundant role for the control of gastrointestinal colonization [39]. Furthermore, a comparative study established that Dectin-1 is essential for both innate and adaptive immune responses to *C. albicans, C. glabrata, C. tropicalis* and *C. parapsilosis*; however, its function in specific responses diverge between the different *Candida* species [40]. Overall, our data show decreased survival and reduced ability of *GNBP3^hades^* fly to clear *C. parapsilosis* cells, but, due to the confusing results described earlier, it is challenging to resolve the real effect of this receptor in *Drosophila* host response in the control of systemic *C. parapsilosis* dissemination.

As a measure of the adult *Drosophila*’s immune recognition process, we examined whether blood cells circulating in hemolymph engulf *C. parapsilosis* cells after septic infection. Representative microscopical pictures demonstrated that phagocytosis of *C. parapsilosis* and *C. albicans* cells occurred *in vivo*. It is interesting that GNBP3 is required for *C. albicans* cells agglutination, prophenoloxidase activation and formation of attack complexes combating this pathogen. All the same time, phagocytosis of *C. albicans* cells was not affected by this sensor molecule’s presence or absence [32]. Meanwhile, these defense functions vary between different *Candida* strains, as *C. glabrata* cells are not agglutinated and they do not entirely trigger the PO cascade in a GNBP3-dependent manner, which occurs with *C. albicans* [16]. Our experiments have limitations as additional elements of the cellular arm of protection in *Drosophila* (e.g. agglutination or PO formation) were not examined. Our results could point to other recognition receptors that might be at play in regulating *C. parapsilosis* infection in *Drosophila* as the Persephone mutant flies were resistant to *C. parapsilosis* and according to a previous research engulfment of *C. albicans* cells was not dependent on the GNBP3 receptor [32]. More detailed studies are needed to obtain deeper insights and decipher the cellular arm of the *Drosophila* immune defense and elimination mechanisms against *C. parapsilosis*.

Taken together, our results demonstrate the importance of a well-functioning *Drosophila* Toll pathway to hinder *C. parapsilosis* infection, and we established the utility of the *MyD88^−/-^ Drosophila* model to analyze differences in the virulence properties of *C. parapsilosis* and related strains.

## Supplementary Material

Supplemental Material

## Data Availability

The authors confirm that the data supporting the findings of this study are available within the article and its supplementary materials.
